# Expressed Beliefs about the Cause of Pain in a Pediatric Population: A Qualitative Study

**DOI:** 10.3390/children10061007

**Published:** 2023-06-02

**Authors:** Laura Menés Fernández, Isabel Salvat, Cristina Adillón

**Affiliations:** Department of Medicine and Surgery, Faculty of Medicine and Health Sciences, Institut d’Investigació Sanitària Pere Virgili, Universitat Rovira i Virgili, 43204 Reus, Spain

**Keywords:** pediatric pain, pain science education, pain perception, child

## Abstract

(1) Background: The aims of this study are to explore what beliefs children and adolescents manifest about the cause of the pain they describe, to compare whether there are differences between beliefs by age and the persistence of pain, and to relate the explanations of the cause of pain with current scientific evidence. (2) Methods: a cross-sectional qualitative study was used. The primary endpoint of the study was obtaining explanations of the cause of pain recorded by means of an open-ended question. The participants were school-age children attending a charted school in the province of Barcelona. (3) Results: The children and adolescents proposed a diverse range of explanations for the cause of pain that they reported in their responses. The most frequent explanation for the cause of pain were pathologies and injuries (45.95%), ergonomic issues (22.60%) and psychological issues (15.95%). (4) Conclusions: There is a lot of variety in the explanations that young people give about the cause of their pain in schoolchildren aged between 10 and 16 years old. There exists a high prevalence of explanations non-associated with tissue damage (ENAD) concerning the causes of pain described. It is necessary that future health prevention programs dedicated to early ages consider which beliefs about the cause of pain are the most frequent in the pediatric population.

## 1. Introduction

People in pain, who are uninformed or incorrectly informed about their pain, have more catastrophic thoughts and fewer adaptive coping strategies compared to people who have correct information [1]. In the case of children, it has been observed that 54% of children believe they are in pain because something “is wrong” [2]. Failure to address these perceptions may influence treatment and facilitate the persistence of pain [3,4].

Persistent pain in childhood is a health problem with an estimated prevalence of 11–54%. In addition, approximately 30% of children experience persistent pain for 3 months or more [5,6,7], and it has been observed that this may also increase the risk of experiencing pain during adulthood [8].

In infancy, persistent pain affects all spheres of a child’s life, having repercussions on social life, performance and school absenteeism [8,9]. This condition can lead to low self-esteem and emotional distress in the child and can also have a negative impact on the child’s family and circle of friends [10,11].

The treatment of persistent pain is multifactorial, and currently, its approach is interdisciplinary in nature [12,13], being an essential part of Education in Pain Neuroscience. The main objective of this approach is to change misconceptions and misbeliefs about pain and its function through an explanatory model [14,15]. This type of intervention has been applied in many situations of persistent pain in adults [16,17,18,19,20,21], and to a lesser extent, in childhood [13,22].

In recent years, several studies have explored children’s and adolescents’ concept of pain through writing, drawings and dialogue sessions [23,24,25]. Other authors have also explored beliefs about pain and coping strategies through interviews or questionnaires [26,27]. In the same vein, recent research has also developed questionnaires to quantitatively assess the conceptualization of pain in childhood and adolescence [28,29]. Inquiring into this conceptualization is particularly useful as it investigates how the child understands what pain is and the processes underlying it. As Pate et al. [24] concluded, understanding children’s prior knowledge of pain is important in the planning of a meaningful pain education program to address persistent pain.

However, in addition to the concept, beliefs about pain and coping strategies, beliefs about what causes pain are fundamental [15]. The meaning and attribution that people give to their painful experience is a fundamental part of the pain itself and of their emotional and behavioral response [30]. However, these beliefs have not been specifically studied in children and adolescents. For these reasons, this study aims (a) to explore what beliefs children and adolescents manifest about the cause of the pain they describe, (b) compare whether there are differences between beliefs according to age and pain persistence, and (c) relate explanations about the cause of pain to current scientific evidence.

## 2. Materials and Methods

### 2.1. Study Design

This is a cross-sectional qualitative study design. The guidelines for conducting qualitative studies established by the Standards for Reporting Qualitative Research (SRQR) [31] were followed. It was conducted by adapting grounded theory [32] and using thematic analysis according to the six steps established by Braun and Clark [33]. The experimental part of the study was conducted between April and June 2021.

The study adhered to the tenets of the Declaration of Helsinki and received ethical approval from the local institutional review board, Ethics Committee for Research with Drugs (CEIm Pere Virgili Health Research Institute) (Ref. CEICm: 114/2018). The study protocol was registered with ClinicalTrials.gov ID: NCT05731401.

As the participants were minors, informed consent was obtained from their legal guardians and verbal assent was obtained from the participants before completing the questionnaire.

### 2.2. Research Team

Three researchers, ranging from novice to expert with respect to their experience with qualitative research, participated in this study. Two of them had PhDs in health sciences, and two of them were involved in clinical activity. Prior to the study, the positioning of the researchers was established, as were the researchers’ beliefs, their prior experience, and their motivation to conduct the research. Our main belief was that young people would come up with a wide range of explanations for the cause of their pain, including explanations unrelated to tissue injury.

### 2.3. Participants

The participants in this study were a convenience sample of schoolchildren aged 10–16 years from a charter school (Barcelona, Spain), a school large enough to have a sufficient sample. The inclusion criteria were as follows: being schooled at the charter school, being between 10 and 16 years old (both included), having or having had pain in the last month, and being able to read and write in Catalan and/or Spanish. Participants were excluded if they had an intellectual disability and/or cognitive impairment that interfered with their participation and if they had not submitted the informed consent form duly completed and signed by their legal guardians. The teachers agreed with the parents or guardians of the participants that they would sign the consent forms, which they did at regular school meetings with the researchers. After consent had been obtained, the participants also provided their assent to participate immediately before they completed the questionnaire. The assent was obtained by the researchers in the presence of the teachers.

### 2.4. Outcomes

The main outcome measures were the explanations of the cause of pain (open response). Demographic information included gender (female/male), age (years) and pain duration (months).

### 2.5. Data Collection

The participants were asked to fill out a questionnaire where personal data (gender and age) and different questions related to pain (presence of current pain or pain in the last month and its persistence) were collected. Finally, they were asked, “Why do you think you have or have had this pain?”.

The questionnaire was administered to schoolchildren who went to the participating school, whose parents had signed the informed consent and who assented to participate just before the questionnaire was administered. In the presence of the teachers, the researchers administered the questionnaire. The researchers and the teachers checked that the students did not comment on each other’s responses.

### 2.6. Data Processing

Participants’ pain belief texts were entered verbatim into the database using Atlas.ti 22. software (Scientific Software Development GmbH, Berlin, Germany). These were checked for accuracy.

The concepts present in the texts were identified and recorded inductively, and then a coding structure was developed in accordance with the above-mentioned objectives of the study using thematic analysis [33].

Themes and sub-themes were developed from the analysis and synthesis of the data.

### 2.7. Data Analysis

The analytical themes were developed inductively and agreed upon in full by the investigators. The issues were discussed by experts in the field, including physiotherapists, academics and methodological experts, at a meeting during the development of the analysis. The investigators followed the 6 stage-analysis provided by Braun and Clark [33]:

(1) Familiarization with all of the data. All of the open-ended responses were read by the researcher team members to obtain a sense of the breadth and depth of the data.
(2) Generating the initial codes. All of the relevant words, phrases or sentences were extracted and given a code, capturing the essence of the meaning. The team discussed, refined and verified all of the codes. The codes were then organized in a data display table.
(3) Searching for themes. The themes were classified by the similar meaning of the codes.
(4) Reviewing the themes. The themes were reviewed by the team and organized in a coherent pattern.
(5) Defining and naming the themes. Each theme was identified by one that captured different aspects of the explanations concerning the causes of pain given by the young people.
(6) Producing the report. The final report that provided a detailed account of each theme was prepared.

For the reliability of the study, triangulation of the researchers was carried out. The trustworthiness of the findings was established through peer debriefing, which was used at several stages of the process as two team members conducted the initial analysis, and a third team member independently validated their conclusions with a reexamination of the data.

## 3. Results

### 3.1. Description of Sample

**Figure 1:**
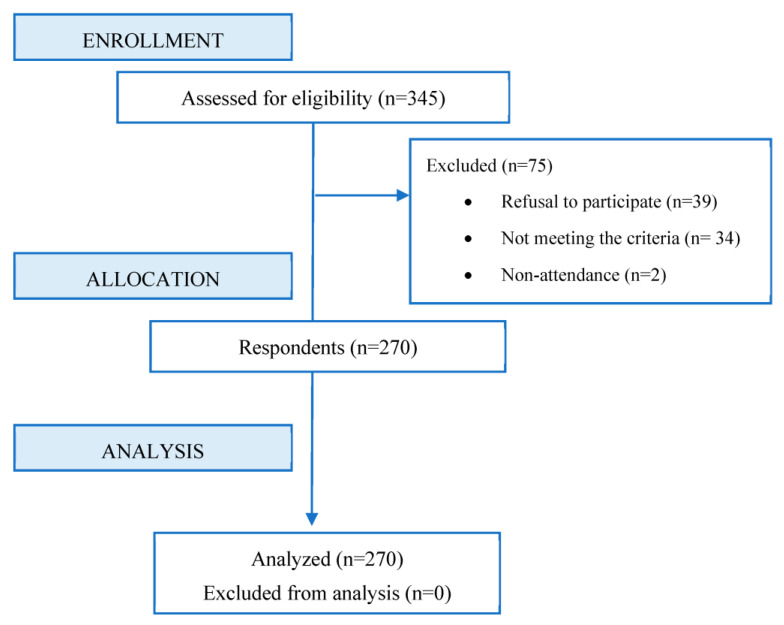
Flow diagram.

Of the 345 potential participants, 306 agreed to participate, of which 25 were excluded for not having or having had pain in the last month, 9 were excluded for having an intellectual disability, and the remaining 2 were excluded for non-attendance (Figure 1).

The final sample size was 270 participants whose mean age was 13.20 (1.82) years, and 50.80% were male (Table 1).

### 3.2. Explanation about the Cause of Pain

Children and adolescents proposed a diverse range of explanations for the cause of their pain that they reported in response to the question, “Why do you think you have or have had this pain?”

The explanations were sub-classified into sub-groups according to the type of explanation. Illustrative quotations for each theme are provided in Table 2.

The most frequent explanation for the cause of the pain was associated with Pathologies and injuries (45.95%).

Three sub-groups were generated from the recurring explanations: injuries (87.90%), wounds (8.87%) and illnesses (3.22%). Most of the participants frequently included the words “wound”, “injury” and “blow” in their explanations and justified the tissue damage by having suffered a contusion, a fall or a burn. On the other hand, some of them provided contextualized explanations justifying the cause of their pain, such as “because I fell off my bicycle” or “because I had an operation for a testicular torsion”. Only 3.48% of the participants referred to illnesses or infections as the cause of their pain. Within the explanations of injury, a distinction was made between those explanations that referred to acute (80.64%) and chronic injury (7.26%). The explanations of the participants classified in the chronic injury subgroup explicitly contain the fact that they were injured long ago. In these explanations, the participants referred to old injuries, which, in certain contexts, they believed were the cause of the pain because they had not healed properly or that there had been something wrong since they had had the injury. In contrast, the participants who explained experiencing acute injuries made no reference to the chronology related to the injury.

Many of the participants explained the cause of pain through ergonomic issues (22.60%). In general, posture is considered to be the first cause of pain, with some participants referring to “having a bad posture” or not “being upright” as being the cause of their pain. While some participants referred to posture in terms of sitting or sleeping position, others commented on the cause of their anatomical posture, such as “because my shoulders are too far forward”. Another frequent ergonomic aspect was the weight of their school backpack. The participants who described the weight of the backpack as the cause of their pain had negative connotations about carrying weight, especially loads on the back. They also tended to mention that “it is too much weight for our back”.

Psychological issues (15.95%) were the second cause described by the participants who reported pain related to emotional states, such as sadness or anxiety. Most of them explained that their pain was a consequence of “being stressed”. Other participants also described stress as a cause of their pain but from the perspective of their personality, describing themselves as “very nervous” or “demanding”.

Around eighteen percent provided explanations associated with development, where growth (9.70%) and, specifically in girls, menstruation (17.30%) were the issues suggested as the causes of their pain. Those participants who suggested growth to be the cause of their pain explained that they were growing too fast, that some structures were growing asymmetrically or that they had “inflammation from growth”.

Menstruation was the explanation for the cause of pain in 24.06% of girls (*n* = 133) over 11 years of age. Some of them clearly stated that it was due to the process of menstruation, while others described it as “things that happen to women” or “my body forces me to go through it because I am not a child anymore”. Furthermore, in many of the explanations, the participants justified the cause of their pain by normalizing the pain in the menstruation process; for example, one participant described, “Because it is something normal for women that happens every month”.

Others described that the cause of the pain was due to ambiental issues (2.00%), such as noise and weather. Those participants who described that noise was the cause of their pain used to refer to localized pain in the head. Some of them accompanied the explanation with emotional language, i.e., the noise made them angry, was unpleasant or “the voices were irritating”. Other participants said that changes in the weather, season or certain weather conditions, especially rain, were the cause of their pain. In these latter explanations, some of the participants described that the weather affected their old injuries or areas of the body that they referred to as their “weak points” or where they were “more sensitive”.

They also explained causes of the pain related to habits (3.00%) in their daily life, such as sleeping or eating. Most of them explained the cause of their pain related to sleep (89%). They gave explanations such as “not getting enough rest” or “not sleeping well”. Only two participants explained their pain in terms of substance issues, specifically food. These participants expressed how they believe that certain types of food were the cause of their pain.

Finally, for a smaller percentage, some of the participants explained the cause of their pain as being related to the pain of someone in their family (1.5%), reporting that it is something normal in their family or that they had the pain because someone in their family also had the same pain. The relative mentioned was usually a first-degree relative, especially the mother.

#### 3.2.1. Explanation about the Cause of Pain According to Age

Younger participants tended to explain the cause of their pain as being associated with pathologies and injuries, while as they grew older, their explanations diversified into other causes beyond injuries. In this sense, the older the participants, the greater the number of explanations not associated with pathologies and injuries. Even so, there were no age differences in terms of the beliefs about the cause of pain in each of the categories. In all of the categories described, there are participants of all ages.

In some categories, there were differences in the explanations based on age. Psychological and ergonomic aspects were found more frequently in participants of 12 years of age. Explanations associated with menstruation were found in girls from the age of 11 years onwards.

The youngest participants (10–11 years old) provided simpler and shorter explanations compared to the older ones. From the age of 12, they indicated their beliefs in the cause of their pain in more detail. For example, while a 10-year-old boy explains that the cause of his pain is “due to growth”, a 12-year-old boy provided a already a more detailed explanation, “Because the bone has grown faster than the muscle and when I make physical effort it hurts, but now that I use insoles it does not hurt so much”.

#### 3.2.2. Explanation about the Cause of Pain According to Pain Persistence

It was observed that most of the children whose pain had been present for longer tended to attribute it to explanations not associated with pathologies and injuries. Thus, it was observed that participants with a pain persistence of more than 3 months had more frequent explanations that did not involve pathologies and injuries.

However, this is not the case in those explanations about injuries where the participants express that they believe that they are “badly healed” and that they have been in pain for a long time.

## 4. Discussion

This is the first study known to the authors to investigate the beliefs that children and adolescents have about the cause of their experiences with pain. Previous studies concerning childhood pain from the child’s perspective do exist. However, most of this research has focused on how the child recounted their pain experience [25,34] and the vocabulary they used [35], explored pain beliefs [27] and coping strategies [26] or the generic concept of “pain” [23]. The present study differs from previous studies in that it does not focus on pain per se but explores the meaning of pain that children attribute to their experiences of pain. Moreover, the prevalence of chronic pain found (33.56%) is similar to that reflected in the literature [5,6].

On the one hand, the most frequent explanation for the cause of the pain was associated with Pathologies and injuries (45.95%). These explanations are consistent with physiology since, after tissue damage, the tissue must undergo remodeling that involves peripheral and central sensitization processes as well as nociceptive facilitation that coexists during the tissue injury to protect it during the healing process [36]. Seven percent of the participants attributed their pain to unhealed wounds or injuries. The children explained situations in which, long after an injury, the tissues should be healed and associated the pain with the injury. However, in the absence of pathology, damaged tissues have a time-delimited healing and remodeling process, and if the perception of pain persists, it should rather be attributed to a maladaptive process of central sensitization [36]. Maintaining the belief that something is not yet healed may itself cause the process of central sensitization and pain perception to persist [4].

On the other hand, several explanations for pain were related to ergonomic aspects (22.60%), with posture being the most frequently cited (67%). According to the participants, incorrect posture, or failure to maintain an upright posture, resulted in their pain. Although this belief is also found in the adult population [37], there is no evidence that maintaining such a posture is beneficial to preventing or treating back pain [38] nor that there is a correlation between pain in different parts of the body and posture [39,40,41,42,43,44], which is the reason why it must be inferred that relating posture to pain is an explanation non-associated with tissue damage (ENAD). On a similar level, although less frequently cited, is the weight of the backpack (31%) as a cause of pain.

Indeed, for decades, it has been believed that the weight and position of a backpack were determinant factors of back pain in children. However, such a relationship is inconclusive [45,46], and the weight of a backpack is not related to musculoskeletal pain in children [47,48]. In fact, according to Goodgold et al. [49], there is a large difference between children’s perceptions of the weight of their backpacks and the actual weight. Additionally, there is also no evidence that the weight and position of a backpack lead to musculoskeletal injuries [50]. These postural alterations produced by carrying the backpack can be considered adaptive, suggesting that the fact of viewing themselves as able to carry the backpack may be more important than the weight of the backpack itself [51,52].

The second most common explanations non-associated with tissue damage (ENAD) were associated with psychological aspects. Stress was the most common explanation. In recent decades, this concept has evolved culturally and socially, no longer referring exclusively to its biological meaning as a life-sustaining response of the organism to adverse situations [53]. While it is true that unresolved stress that persists over time may favor the lowering of pain thresholds, it is not stress itself that is the main cause of perceptions of pain [54].

Something similar occurs in terms of rest, which was the most popular explanation relating to habits. People who have difficulty sleeping or who feel that they are poorly rested tend to be more sensitive to mechanical stimuli and experience pain more easily [55]. However, the causal relationship between the association between rest and pain and the mechanisms underlying it are unclear [56,57].

Along the same lines, 22% of the participants explained the cause of their pain in terms of development issues. Twenty-six percent of them reported growth as the cause of their pain. During childhood, pain in the extremities is on the list of the most frequent pains. Given this clinical picture, if it is concluded that there is no pathology that could justify the perceived pain, a diagnosis of growing pain is made [58]. Currently, there is no evidence that growing up produces nociception and, therefore, pain associated with growing up belongs to non-inflammatory musculoskeletal pain syndromes. These syndromes are not related to structural or tissue pathologies but rather to a sensitization process [59]. It should be considered that, in growing pain, growing up has no relation to pain and that it is an amplification response to pain during childhood [60,61]. Moreover, 17% of the female participants older than 11 years reported menstruation as the cause of their pain. The normalization of pain during menstruation is common among young women and is closely related to the conceptualization of this process in the family and friendship circle [62]. Pain associated with menstruation in the absence of gynecological pathologies is termed primary dysmenorrhea [63,64] and is now considered to be a chronic pain condition [65], which is a consequence of an increase in the concentration of prostaglandins [66]. However, it should be noted that social learning and negative expectations induced by a nocebo are closely related to the production of prostaglandins [67].

Two percent of the participants reported ambiental issues as being the cause of their pain. (2.00%), such as noise and weather. Noise is defined as a confusing mixture of sounds. Sound is the brain’s interpretation of the frequency, amplitude and duration of sound waves that reach the ears. In terms of physiology, what the individual understands as noise is nothing more than the brain’s construction of sound energy. Several studies have found that in people who identify noise as a trigger of their pain, exposure to it generates painful perceptions [68]. For this reason, several authors have stated that there is an association between environmental noise and pain, especially in headaches [69,70]. However, the reason for this association is not clear [71]. Recurrent headaches following noise may be the consequence of a sensitization process resulting from individuals trying to avoid the stimulus they perceive as causing headaches. In this case, noise exposure would be associated with pain but would not be the cause of the pain, instead being a learned trigger [72,73].

Some of the participants believed that weather is the cause of their pain, especially rainy conditions. Despite the fact that these beliefs are very common [74], the association between climate and pain has been difficult to characterize [75]. Although there is current evidence concerning how people in pain can be more sensitive to weather, it could be considered that this is due to sensitized nociceptors and reduced descending inhibitions [76]. Thus, explanations for the cause of pain associated with noise and weather could be considered explanations not associated with tissue damage (ENAD).

Some of the participants explained their pain in terms of pain in their families (1.5%). Social or vicarious learning influences pain perception in laboratory and clinical settings, such as abdominal pain, in both adults and children [77]. Several studies have described the association between first-degree relatives with chronic pain and the development of chronic pain in their children [78,79]. It is likely that modeling plays a role in the phenomenon of pain-prone families, which would explain why children have been observed to show pain syndromes identical to those of their parents rather than those present in their childhood [80].

Furthermore, explanations not associated with pathologies and injuries were more common in children who reported having pain for more than a 3-month duration. It is likely that when pain persists and cannot be associated with harmful events, other explanations are sought, sometimes generating beliefs that could encourage the perpetuation of pain. Moreover, explanations not associated with pathologies and injuries were found more frequently with increasing age. It seems that the older participants have the idea of pain being a multifactorial experience and not only linked to tissue damage in contrast to the younger ones. Youths appear to develop a more nuanced understanding of pain as they age. However, it seems that most of the explanations given by young people could be related to cultural influences. Learning plays a fundamental role in the perception and interpretation of symptoms. The meaning of pain is learned within a culturally determined framework over the years where the family and social environment are crucial [81]. This could also explain why more participants with explanations not associated with tissue damage about the cause of pain were found in those who were older.

At this point, this study has some limitations. The first is that the beliefs found may not be extrapolated to other populations of the same age range. In addition, as this was a school-based study, it was not possible to obtain medical information about whether the pain described had a biological basis. Another limitation of the study is that only the beliefs of the schoolchildren were studied through the pain they themselves described. It is possible that many of the beliefs explained by the participants are held by others who did not report them because they were different from their actual experience of pain. Finally, it can be considered a limitation of the study that no methodological triangulation was performed, as the beliefs about the cause of their pain were only analyzed in one way. Therefore, it is possible that no relevant information has been obtained on the topic analyzed in this study.

A potential clinical implication of this research is that it reveals the most common beliefs of schoolchildren aged 10–16 years from a direct-grant school. Knowledge of these beliefs is essential to develop better pain education programs for the pediatric population. Future research should explore how to change these beliefs and how this can influence the perception of pain in children and adolescents.

## 5. Conclusions

In summary, there is a lot of variety in the explanations that young people give about the cause of their pain. There is a higher percentage of explanations for the cause of pain described as being unrelated to tissue damage events compared to explanations associated with tissue damage (EAD) in children and adolescents aged 10–16 years. Explanations not associated with tissue damage (ENAD) concerning the cause of pain are more frequent in older children and result in the greater persistence of pain.

## Figures and Tables

**Table 1:** Descriptive characteristics of participants.

Characteristics	*n* (%)
Gender	
Female	133 (49%)
Male	137 (51%)
Age (years)	
10	16 (6%)
11	43 (16%)
12	41 (15%)
13	42 (16%)
14	51 (19%)
15	44 (16%)
16	33 (12%)
Location	
Head	112 (41%)
Upper limb	147 (54%)
Lower limb	161 (60%)
Abdomen	91 (34%)
Back	127 (47%)
Multiple	169 (63%)
Pain duration	
Less than 1 month	101 (37%)
1–3 months	70 (26%)
More than 3 months	99 (37%)

**Table 2:** Quotations to illustrate themes and sub-themes classified according to explanations.

Pathologies and Injuries		
Injury	Acute	“Because I broke my finger and had to wear a plaster cast” (girl, 10 years old with pain in their hand; pain lasting less than 3 months). “I got the ball kicked in my belly playing football” (boy, 14 years old with abdominal pain; pain lasting less than 3 months).
	Chronic	“Because three years ago I was jumping in the “salting”, and I think I did a bad posture and I think muscle moved out of place.” (boy, 12 years old with chest pain; pain lasting more than 3 months) “Because a few years ago I broke a bone in my wrist, and it did not heal properly. (girl, 14 years old with wrist pain; pain for more than 3 months)
Wound	“I was playing, and I started running and I slipped and got a scratch” (boy, 12 years old with knee pain; pain lasting less than 3 months). “Because I fell down the stairs and they had to put eight stitches in my head” (boy, 14 years old with headache; pain lasting less than 3 months).	
Disease	“Because I got out of the pool without covering up and I caught a cold” (girl, 10 years old with headache; pain lasting less than 3 months). “Why I had COVID-19” (boy, 12 years old with generalized pain; pain lasting less than 3 months).	
Ergonomic issues		
Posture	“Because I sleep in the wrong posture”. (boy, 11 years old with back pain; pain lasting less than 3 months) “Because I have bad posture and I forget to stand up straight when I am sitting in class”. (girl, 16 years old with head and back pain; pain lasting more than 3 months).	
Backpack weight	“The one in the back [referring to pain] because of the weight of the backpack and the one in the head has always happened to me, but I do not really know why”. (girl, 11 years old with head and back pain; pain lasting less than 3 months) “Because the backpack weighs a lot and sometimes it weighs so much that my back cannot hold much and I hurt myself.” (girl, 14 years old with back pain; pain lasting less than 3 months)	
Developmental issues		
Menstruation	“My gut hurts because of my period and my head hurts because of the same thing. (girl, 12 years old with abdominal pain and headache; pain lasting less than 3 months). “Lower abdominal and back pain are normal menstrual pain in women, hormone changes...” (girl, 16, headache with abdominal and back pain; pain lasting more than 3 months).	
Growth	“Because the bone has grown faster than the muscle and when I do physical effort it hurts. However, now that I wear insoles it does not that much”. (boy, 12 years old with foot pain; pain lasting less than 3 months) “I have pain due to growth because it’s been a year that I have been growing 1 cm per month”. (boy, 14 years old with pain in upper and lower extremities; pain for more than 3 months).	
Psychological issues		
Stress	“It happens to me because of the accumulated stress of some things.” (boy, 11 years old with neck and back pain). “Because I unload all the nerves in these areas, and as I am a very nervous person...” (girl, 13 years old with back pain; pain lasting more than 3 months)	
Emotions	“Because of the emotional load I carry, which I hold in my back. In addition, the head also because of stress, lack of sleep... I am not sure about the knees, maybe it’s the same thing...” (girl, 14 years old with headache and back and knee pain; pain for more than 3 months). “Because of what my friends do to me, I do not know why, I have not done anything to them. (girl, 10 years old with headache; pain for more than 3 months)	
Habits		
Rest	“My head hurts because I do not rest for the right amount of time”. (boy, 14 years old with headache; pain lasting less than 3 months). “My head hurts because I do not sleep much”. (boy, 16 years old with head and back pain; pain for more than 3 months).	
Feeding	“Because I drank milk at night” (girl, 11 years old with headache; pain lasting less than 3 months). “It’s because I like chocolate and it makes my head work too fast and it gets tired and sore” (boy, 10 years old, headache; pain for more than 3 month).	
Environmental issues		
Noise	“Most of the things I have are the unbearable noises and irritable voices.” (boy, 13, with headache and back pain; pain for more than three months) “Three things always hurt me: my head hurts when there are loud noises, the belly from my period and my back from the weight I carry in my backpack”. (girl, 14 years old with headache, abdominal and back pain; pain lasting less than 3 months)	
Weather	“Because when the weather changes it affects my head, it’s my weak point” (boy, 14 years old with headache; pain for more than 3 months). “Because I am more sensitive when it rains” (girl, 15 years old with headache and back pain; pain for more than 3 months).	
Social family influence		
“I have this pain because I have something my mother had, which is that your legs are kind of closed and you have to wear insoles. (boy, 11 years old with pain in lower limbs; pain lasting less than 3 months) “Because my mother also has this pain.” (girl, 11 years old with headache; pain for more than three months). “Because it happens to all of us in my family when we get nervous, it affects our head. (girl, 14 years old with headache; pain lasting less than 3 months)		

## Data Availability

The study did not report any data.
